# Evaluation of Polish Wild Mushrooms as Beta-Glucan Sources

**DOI:** 10.3390/ijerph17197299

**Published:** 2020-10-06

**Authors:** Iwona Mirończuk-Chodakowska, Anna Maria Witkowska

**Affiliations:** Department of Food Biotechnology, Faculty of Health Sciences, Medical University of Bialystok, Szpitalna 37, 15-295 Bialystok, Poland; witam@umb.edu.pl

**Keywords:** wild edible mushrooms, β-glucans

## Abstract

Mushroom beta-glucans show immunomodulatory, anticancer and antioxidant features. Numerous papers have been published in the last years on fungal polysaccharides, especially beta-glucans, demonstrating their various biological activities. However substantial data about beta-glucan contents in many mushroom species, especially wild mushrooms, are still missing. Therefore, the main objective of the study was to evaluate β-glucans in 18 species of wild mushrooms and three species of commercial mushrooms for comparison purposes. The contents of β-glucans were determined by the Megazyme method and with the Congo red method, which differ in analytical procedure. Among wild mushrooms, the highest mean β-glucan content assessed with the Megazyme method was found in *Tricholoma portentosum* (34.97 g/100 g DM), whereas with the Congo red method in *Lactarius deliciosus* (17.11 g/100 g DM) and *Suillus grevillei* (16.97 g/100 g DM). The β-glucans in wild mushrooms assessed with the Megazyme method were comparable to commercial mushrooms, whereas β-glucans assessed with the Congo red method were generally higher in wild mushrooms, especially in *Russula vinosa*, *L. deliciosus* and *S. grevillei.* This study indicates wild mushrooms as interesting material for β-glucan extraction for food industry and medicinal purposes.

## 1. Introduction 

Glucans are polysaccharides composed of glucose monomers, present in the cell walls of bacteria, algae, plants, as well as yeasts, micro- and macrofungi. Despite many differences between the composition and organization of mushroom cell walls, there are some solid elements that form the core scaffold. The main saccharide components of mushroom cell walls are chitin; α-glucans; and β-glucans, mainly 1,3–1,6-β-d-glucans and others like linear 1,3-β-d-glucans and linear and branched 1,6-β-d-glucans [1,2,3,4,5]. Some of these polysaccharides, especially triple-helix 1,3–1,6-β-d-glucans, show a number of proven therapeutic properties, including immunomodulatory, anticancer and antioxidant features [6,7,8,9,10]. Besides glucans, more complex polysaccharides occurring in mushrooms are heteropolysaccharides such as heterogalactans, heteroglucans and heteromannans. Heteropolysaccharides are characterized by extra variability on their monosaccharide composition, anomeric configuration, branching and linkage type [11].

β-glucans are β-d-glucose polysaccharides. The main criterion for the classification of these compounds is their structure. For example, the type of glycosidic bond connecting glucose monomers, the degree of chain branching, and the length of side chains [2]. Among mushroom β-glucans, there are mainly branched glucans, where β-d-glucose molecules are connected by β-1,3 bonds and have short and numerous side chains with β-1,6-link coming off the β-1,3-backbone [2,8,12,13,14]. Mushrooms 1,3–1,6-β-d-glucans in literature are often shortly labeled as β-glucans [10].

Beta-glucans are not synthesized by the human body; therefore, they are recognized by the immune system. The activity of mushroom beta-glucans may vary depending on the type of monomers that build the structure of these compounds, the size of the molecule, the degree of its branching and solubility in water, as well as the structure that beta-glucans form in the presence of water. Studies show that high molecular weight molecules with β-1-3- bonds in the backbone have the best anticancer properties [15,16]. The majority of β-1,3-glucans show resistance to gastric juice. In unaltered form, they pass into the small intestine, where they bind to macrophage Dectin-1 receptors in the intestinal wall and then are transported to the spleen, lymph nodes, and bone marrow. In macrophages, high molecular weight β-glucans are degraded into smaller fragments, which are then bonded by Complement Receptors 3 (CR3) on immune system cells, including granulocytes. Thus, the immune response against cancer cells is stimulated [16,17]. The great diversity in the structure of the beta-glucans affects their various biological activities. The scientific reports to date attribute beta-glucans immunomodulating, anti-cancerogenic, hypolipemic, hypoglycemic and protective properties to the circulatory system [18,19]. One of the most widely studied biological activities of plant and mushroom beta-glucans is hypocholesterolemic activity. Although, many of health promoting activities are connected to their effect on immune system, this property seems to be related to their ability to reduce cholesterol absorption and to scavenge bile acids [20].

The content of beta-glucans in mushrooms depends on the species, growth environment, and mushroom maturity. Beta-glucans isolated from macrofungi are considered to be natural modifiers of immune response, i.e., biological response modifiers (BMR). The beta-glucan molecules of the individual mushroom species differ in the structure of the backbone, the number and type of chemical bonds, as well as the type and number of side chains, structure (e.g., triple helix, single helix or random helix) and molecular weight. According to current knowledge the conformation of the β-glucans is a determining factor of their bioactivity [9,10,21,22,23,24].

Mushrooms with high β-glucan content have a higher nutritional, pharmaceutical and economic value. They are used in food, dietary supplement and medicine industry [22,25,26,27,28]. There are few publications only on the quantitative evaluation of β-glucans [9,29,30], and existing reports focus mainly on a limited number of cultivated mushrooms species [31,32,33].

In recent years some new methods were developed for the determination of β-glucans, among others, the enzymatic method (Megazyme, Ireland) for the determination of β-glucans in mushrooms and yeast [14,30] and Congo red method, which enables to detect β-glucans with triple helical structure [9,29,34,35]. Therefore, the main objective of this study was the quantitative evaluation of β-glucans in wild and commercial mushrooms used for comparison purposes, with the two analytical methods. According to our knowledge, this is the first study that evaluates the content of β-glucans by two methods the Megazyme method and the Congo red by Nitschke et al. [29] in 21 edible mushroom species including 18 wild mushroom species.

## 2. Materials and Methods

### 2.1. Studied Materials

The research material consisted of 21 species of edible mushrooms (between three and four samples of each species), including 18 wild and three commercial cultivated species, for comparison purposes. In total, 68 mushroom samples were investigated.

Wild species were harvested from an area of seven municipalities of the Podlaskie Voivodeship (northeastern region of Poland). The exact location of the sampling area and physiographic characteristic of the study area were described earlier in [36]. The fruiting bodies of the mushrooms were classified using atlases and identification keys as described in [36]. The studied wild mushroom species were *Armillaria mellea* (Vahl) P. Kumm, *Boletus edulis* Bull., *Boletus subtomentosus* (L.), *Cantharellus cibarius* Fr., *Cortinarius caperatus* (Pers. Fr.), *Imleria badia* (Fr.) Fr., *Lactarius deliciosus* (L.) Gray, *Leccinum aurantiacum* (Bull.) Gray, *Leccinum scabrum* (Bull.) Gray, *Macrolepiota procera* (Scop.) Singer, *Suillus bovinus* (Pers.) Roussel, *Suillus grevillei* (Klotzch) Singer, *Suillus luteus* (L.) Roussel, *Tricholoma equestre* (L.) P. Kumm., *Tricholoma portentosum* (Fr.) Quel., *Xerocomellus chrysenteron* (Bull.) Šutara., *Russula heterophylla* (Fr.) Fr. and *Russula vinosa* Lindblad. All samples were collected from different locations (not to originate from one mycelium) and far from transportation routes and other pollutants.

Commercial cultivated mushrooms were purchased in local markets: *Agaricus bisporus* (J.E. Lange) Imbach, *Lentinula edodes* (Berk.) Singer. and *Pleurotus ostreatus* (Jacq.) P. Kumm. Each of the three samples came from a different batch for each species.

The collected fruiting bodies (200–300 g) were classified using mushroom atlases and identification keys [37,38]. This was followed by manual removal of debris, such as leaves, snails, insects, and soil. Next, the mushrooms were cut into smaller pieces using disposable plastic knives.

### 2.2. Sample Preparation Procedure

After cutting, prepared fruiting bodies were weighted for about 100 g per sample of the edible parts (stem and pileus). Then, they were stored frozen (−70 °C) in grip seal bags until freeze-drying. Next, the samples were freeze-dried at −50 °C under reduced pressure of 0.027 mBar using the FreeZone FreezeDry System (Labconco, Kansas City, MO, USA) to a solid dry mass (approx. 36 h). Mushroom samples were weighed before and after the drying process on an analytical scale with an accuracy up to 0.01 g. The freeze-dried mushrooms were ground in a ceramic mortar and stored in a desiccator until analysis in sealed grip bags without air. Next, the samples were extracted for further determination of β-glucans with the Megazyme method and the Congo red method, as described in [39].

### 2.3. Glucan Determination

#### 2.3.1. The Megazyme Method

β-glucans were determined using the K-YBGL β-glucan Assay Kit (Yeast and Mushrooms) (Megazyme, Bray, Ireland). This enzymatic test is a quantitative method to determine 1,3-1,6-β-d-glucan in mushroom and mycelial products, yeast and fungal preparations [14].

The assay involved an initial hydrolysis of the glucans in concentrated hydrochloric acid, followed by quantitative hydrolysis using enzymes and spectrophotometric determination of unbound d-glucose. d-glucose content is a measure of total glucan (α-glucan + β-glucan + d-glucose from oligosaccharides, sucrose + free d-glucose content. Determination of total glucan content was described previously in [39]. The α-glucan solubilization, hydrolysis and measurement were performed as described in [39]. The β-glucan content of the studied mushrooms was calculated from the difference of total glucans (α-glucan + β-glucan + d-glucose from oligosaccharides and sucrose + free d-glucose) and α-glucans + d-glucose from sucrose and free d-glucose.

Absorbance measurements for the tested samples were performed using UV-1800 Shimadzu spectrophotometer (Shimadzu, Kyoto, Japan). Absorbance was converted into μg of d-glucose using conversion factor (F), calculated according to the instructions in the kit. A detailed description of glucan determination was published previously in [39].

According to the manufacturer’s instruction from 2019, the hydrolysis of glucans can be alternatively performed with sulphuric acid and with hydrochloric acid. McCleary and Draga [14] found that hydrolysis in sulphuric acid or hydrochloric acid gave similar results for most mushroom samples [14].

#### 2.3.2. The Congo Red Method

According to Nitschke et al. [29], the Congo red method is suitable for the determination of triple helical structures of 1,3-1,6-β-d-glucans. The lyophilizates were subject to a three-stage extraction in hot hydrochloric acid (0.6 mol/L HCl), as well as sodium (1 mol/L NaOH) and potassium hydroxide (1 mol/L KOH). Five hundred mg of each sample was used for extraction. The extraction was carried out in accordance with Nitschke et al. [29]. After neutralizing the obtained fractions, β-glucan content was determined spectrophotometrically using their ability to form colored complexes with Congo red. The absorbance values were referred to the schizophyllan standard curve and converted into g/100 g dry mass. Absorbance measurements for the tested samples were performed using UV-1800 Shimadzu spectrophotometer (Shimadzu, Kyoto, Japan). A detailed description of β-glucan determination was published previously in [39].

### 2.4. Statistical Analysis

Statistical analysis was performed using Statistica 12 software (StatSoft, Inc., Tulsa, OK, USA). The normality of distribution verified, using the Lilliefors (Kolmogorov–Smirnov) test and the Shapiro–Wilk test, showed no normal distribution of the analyzed quantitative variables; therefore, non-parametric tests were used for comparison. The Mann–Whitney U test was used to compare the content of β-glucans assayed by the Megazyme method and by the Congo red method between wild growing and commercially cultivated mushrooms, as well as to compare various extraction fractions of β-glucans in the Congo red method.

## 3. Results and Discussion

### 3.1. Evaluation of the Total Glucan, Alpha-Glucan and Beta-Glucan Content Using Megazyme Assay Kit

The study of 68 samples of edible mushrooms showed high variability in the content of the analyzed components in the studied mushroom species. The moisture content of the analyzed samples ranged from 82%–95% (Table 1). The highest moisture content was found in *Suillus grevillei* (92.4%–95.7%) and the lowest in *Macrolepiota procera* (82.0%–87.1%) and *Suillus bovinus* (82.9%–85.7%). These values are in accordance with other studies [40,41,42].

The same methods of extraction and determination of the total content of glucans, α-glucans, and β-glucans used in our study was also used by other authors [3,30,43,44]. The tested 21 commercial and wild grown mushrooms showed results that are in accordance with the studies of Sari et al. [30] and McCleary and Draga [14], who also studied several mushrooms species with the Megazyme assay kit.

Table 1 presents the total content of glucans, α-glucans, and β-glucans in the studied mushrooms. Total glucan (α-glucan+ β-glucan+ d-glucose from oligosaccharides and sucrose, and free d-glucose) content in mushrooms ranged from 11.4 g/100 g DM in *Macrolepiota procera* to 45.9 g/100 g DM in *Pleurotus ostreatus*.

The α-glucans + d-glucose from sucrose+ free d-glucose in wild mushrooms ranged from 0.70 g/100 g DM in *Suillus grevillei* to 8.57 g/100 g DM in *Russulla heterophylla*. This is in line with previous studies, where the α-glucan content in cultivated and wild mushrooms was usually less than 10 g/100 g DW [14,30,43].

The β-glucan content of the wild mushrooms ranged from 10.50 g/100 g DM in *Macrolepiota procera* to 34.97 g/100 g DM in *Tricholoma portentosum* (Table 1). Among the commercial mushrooms, the lowest β-glucan content was found in *Agaricus bisporus* (11.36 g/100 g DM) and the highest in *Pleurotus ostreatus* (40.34 g/100 g DM) (Table 1).

### 3.2. Evaluation of Beta-Glucans Using Triple Stage Extraction and the Congo Red Method 

The content of β-glucans in the studied wild mushrooms ranged from 1.95 g/100 g DM in *Cantharellus cibarius* to 17.11 g/100 g DM in *Lactarius deliciosus*. While in commercial mushrooms, it ranged from 3.12 g/100 g DM in *Agaricus bisporus* to 12.39 g/100 g DM in *Pleurotus ostreatus* (Table 2).

β-glucan triple extraction method using sodium and potassium hydroxide solutions as well as hydrochloric acid solution was used. This method of extraction and colorimetric determination of β-glucans was validated by Nitschke et al. [29] and Semedo et al. [9]. Nitschke et al. [29] found that this method indicates triple helix 1,3-1,6-β-d-glucans, and alkaline solvents are more effective to extract highly branched triple helical β-glucans, what was also confirmed by Ogawa et al. [34], Mao et al. [35], Smiderle et al. [45], Morales et al. [4], and de Jesus et al. [46].

The triple-helix β-glucans with high molecular weight show stronger anticancer properties [10,15]. Mushroom glucans often form intermolecular interactions yielding complex polymers that are difficult to isolate, but recent studies suggest a simple and effective procedure that allows to separate different glucan structures. de Jesus et al. found that, linear 1-3-α-d-glucans are insoluble in diluted aqueous NaOH solutions, whereas 1,3-1,6-β-d-glucans are soluble [46].

**Figure 1:**
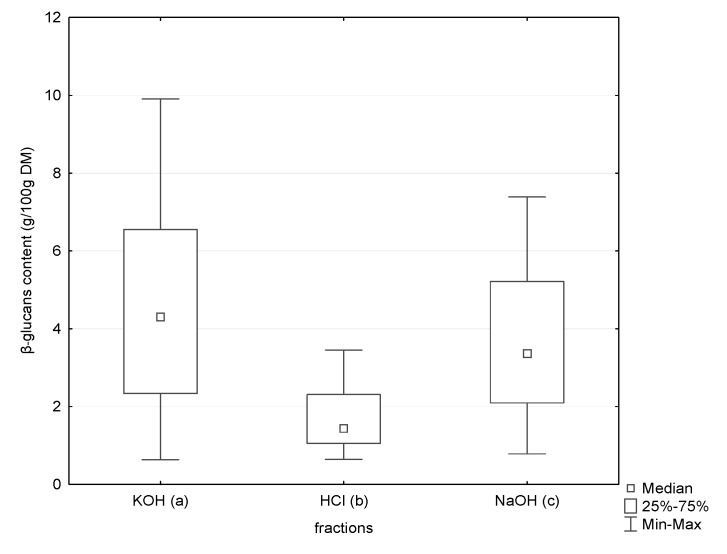
Content of β-glucans in three tested fractions (KOH, HCl and NaOH) using the Congo red method by Nitschke et al. [29]; *p* a/b < 0.0001; *p* b/c < 0.0001; *p* a/c < 0.0001.

The triple-helix β-glucans are considered to be soluble in alkaline solutions [3,15]. Among β-glucans found in mushrooms, there are compounds of different molecular weights. Molecular weight is one of the factors influencing their solubility. Therefore, to isolate β-glucans with a triple-helical structure, three-stage extraction was applied. According to the literature, β-glucan molecules with a high molecular weight show better solubility at an alkaline pH, whereas molecules with a low molecular weight at an acidic pH [29]. In our study, we observed a statistically higher content of β-glucans in the alkaline extracts (Figure 1).

The triple helical structure of β-glucns depends on the degree of their branching. β-glucans with few or without β-1,6-linkages usually have a single helix structure, but 1,3-β-d-glucans with multiple β-1,6-linkages can form triple helical structure [29]. In the Congo red method, the dye interacts with the triple helix of 1,3-1,6-β-d-glucans and does not react with other polysaccharides as chitin, chitosan or glycoproteins [29]. The amino groups of the dye of Congo red link to hydroxyl groups of the β-glucans chain by hydrogen bonds [9]. The Congo red solution after reaction with the Congo red positive β-glucans gives a bathochromic shift, which can be determined spectrophotometrically [29]. Nitschke et al. [29] found that Congo red binds only Congo red positive β-glucans with the triple-helix structures. However, it should be stressed that the triple helical structures of β-glucans are susceptible for conformation transition in some solvents and under some chemical and physical conditions like very high temperature [10].

### 3.3. Extraction and Quantitative Determination Methods of β-Glucans

The current literature on the methods of extraction [47,48,49] and determination of β-glucans is very differentiated. For the extraction of β-glucans, the following procedures are used:

- Extraction with water at different temperatures;
- Extraction in aqueous solutions of different pH;
- Extraction under special physical conditions, e.g., using ultrasound, microwave, radiation or increased pressure [50].

The most common methods to obtain mushroom β-glucans are procedures with hot water extractions. Although, β-glucans with complex conformation frequently need more aggressive extraction with hot alkali solutions, concentrated sulphuric acid or chloroacetic acid [50].

The extraction procedures are multistage and consequently lead to degradation of the cell wall with the release of its structural components, which include β-glucans, among others [47,50].

In our study, the Megazyme procedure [14] and the three-stage procedure extraction in alkaline and acid water solutions according to Nitschke el al. [29] for beta-glucans were performed.

Various methods for the quantitative determination of β-glucans are described in the literature. Among them, the following can be distinguished: the immunoenzymatic method (ELISA) [51], the fluorimetric method using aniline blue [29,52], the spectrophotometric method with enzymatic hydrolysis [53,54,55] or the spectrophotometric method using Congo red [29], high-performance liquid chromatography (HPLC) [56] and others like Nuclear Magnetic Resonance (NMR) [57].

The immunoenzymatic method (ELISA) is based on the use of antibodies specifically reacting with antigens. In the case of Mizuno et al. [51], solutions of 1,3-1,6-β-d-glucans—lentinate and grifolate—were used to produce antibodies. The obtained antibodies were used to determine these β-glucans in several species of mushrooms. The immunoenzymatic method is characterized by high limitations resulting from the specificity of the antibodies, which show high specificity for binding the antigens used for immunization. Some 1,3-1,6-β-d-glucans are not recognized by such antibodies produced in this way, which may result in underestimation [9,51]. In our study, the results for β-glucans were higher by 1–2 orders of magnitude (Table 1 and Table 2) with reference to Mizuno et al. [51].

The fluorimetric method for the determination of 1-3-β-d-glucan content using aniline blue was used for the first time for the determination of the content of these compounds in food samples by Ko et al. [52]. For the first time, Nitschke et al. used the fluorimetric method for the determination of 1,3-β-glucans of a non-branched structure in mushrooms [29] as well as Gil Ramirez et al. [50]. Some studies declare that such fluorimetric method is specific to (1→3)-β-d-glucan binding, but it showed certain limitations that were not always appraised, i.e., the polymerization degree, presence/absence of substituents and their chemical conformation might modulate the fluorimetric determination [8,50,58].

The spectrophotometric enzymatic method was developed by McCleary and Holmes [59]. It is based on enzymatic hydrolysis of polysaccharides to release d-glucose and determination of its content by spectrophotometric method. It was first applied for the determination of β-glucans in cereal grains by McCleary and Holmes [59]. After slight modification by Manzi and Pizzoferrato [60], it was used for the determination of β-glucans in fungi. In later years, the determination of β-glucan content by enzymatic method in fungi was carried out by Bak et al. [61], Cha et al. [53], and Kang et al. [54]. This enzymatic method with modifications is currently recommended by the American Association for Clinical Chemistry (AACC). It allows for accurate and precise determination of polysaccharides and indirect (computational) assessment of β-glucan content. The β-glucan content is calculated from the difference between total glucans (α-glucans + β-glucans) and α-glucans. The method can be used for the determination of both linear and branched β-glucans. It can also be used to assess the β-glucan content in the presence of other polysaccharides, i.e., α-glucans or cellulose [14,62].

In our study, an enzymatic method Megazyme [14] was used to assess the β-glucans content in mushrooms and the results obtained (Table 1) were similar to those presented by other authors who used this method [30]. Much lower β-glucan content was found by Manzi et al. [63], which most likely resulted from the presence of chitin attached to the main glucan chain, resulting in accessed of the enzymes to the glucan chain. This assumption can be confirmed by the fact that the β-glucan content of mushroom species in the Manzi et al. study increased significantly after thermal treatment, which most probably contributed to the partial degradation of bonds between chitin particles and glucan chains [63]. As stated by other authors, the Megazyme method seems to give higher results compared to other methods [55,64].

The interaction between Congo red and β-glucans of plant origin has been known for decades [34]. However, it was only in 2007 that Mao et al. [35] using a curdlan, non-brnched 1,3-β-d-glucan, found that that Congo red binds to the triple helical chain β- glucans. Exposure of curdlan to Congo red did not result in enhancing the solution color [35]. In 2011, Nitschke et al. [29] determined triple-helix 1,3-1,6-β-d-glucans by the spectrophotometric method using Congo red in the mycelia cultivated in vitro and in fruiting bodies of edible mushrooms, thus confirming the ability to bind triple–helix 1,3-1,6-β-d-glucans to this dye [29]. The specific ability of triple-helix β-glucans to bind Congo red dye particles has also been used for determination by Semedo et al. [9] and Morales et al. [4]. In our study, the content of β-glucans in mushrooms was therefore evaluated by spectrophotometric method using Congo red dye. The results obtained (Table 2) were within the range of values presented by other authors [9,29].

Toledo et al. [56] used high-performance liquid chromatography (HPLC) and a spectrophotometric method to compare both quantitative methods for the determination of β-glucans, finding no statistically significant differences between the methods used [56]. It can therefore be concluded that the spectrophotometric method is as precise as the HPLC method, but cheaper, faster and easier. Considering these features, the spectrophotometric method can be selected as the first-choice method in the evaluation of β-glucan content in mushrooms [56]. The content of β-glucans in our research was determined using the spectrophotometric method, and the values of β-glucans obtained were of the same order of magnitude (Table 1) compared to the results obtained by Toledo et al. [56].

The NMR spectroscopy is useful for the investigation of glucan conformation and gives information on the anomeric configuration and ring conformation, position, and proportion of branching. This method is accurate, but data interpretation is complicated [8,57].

In our study, the much simpler and cheaper Congo red method was used, which according to Nitschke et al. [29] allows to detect the triple-helix conformation of β-glucans by examining the visible spectrum of its complex with dye [29,35]. The Congo red method was used for determination of triple helical glucan structure because of its proven interaction with triple helical structures of beta-glucan without the need of extensive clean–up [29,35,65]. Moreover, current study by Morales et al. [4], which used advanced NMR research techniques, confirms that the Congo red colorimetric assay indicates tridimensional conformation in mushrooms beta-glucans [4].

The Megazyme method, despite its numerous advantages, does not allow to determine the triple-helix conformation of beta-glucans. Therefore, a second analytical method, e.g., NMR is required to determine a conformation structure of this polysaccharides [3,5].

**Figure 2:**
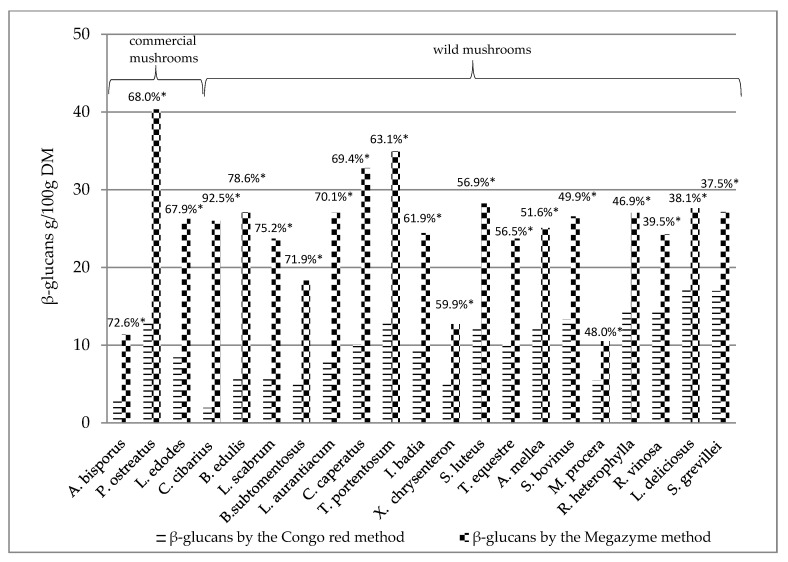
Comparison of β-glucan contents assayed with the Congo red method and with the Megazyme method, expressed as the percentage difference. * Percentage difference between beta-glucans assayed by the Congo red method in relation to beta-glucans assayed by the Megazyme method, given in order of decreasing value of percentage difference.

The determined β-glucans by the Megazyme method and the Congo red method of individual mushroom species were collated to determine the percentage difference between beta-glucans assayed with Congo red dye and the Megazyme method (Figure 2). It was found that the Megazyme method gives higher values than the Congo red method, in general. The Megazyme methods gives the higher results compared to other methods what was described by Gründemann et al. [55].

The results in Figure 2 are presented according to the decreasing percentage difference of beta-glucans determined by the Congo red method as compared to beta-glucans determined by the Megazyme method. The highest percentage difference between β-glucans detected by the Congo red method and by the Megazyme method was found in *Cantharellus cibarius* (>90%) (Figure 2), while the lowest percentage of β-glucans by the Congo red method was found in *Suillus grevillei*, *Lactarius deliciosus* and *Russula vinosa* (<40%) (Figure 2).

The lower values found in the Congo red method may be related to the fact that triple helical structure of β-glucans can be detected with this method [4,9,29]. It is known that mushroom β-glucans can have various conformations [10], which may not be detected using Congo red. On the other hand, the extraction method might not extract all triple helical β-glucans. Moreover β-glucans can adopt triple helical chain conformation in water solutions and form other structures in polar solutions for example [10].

The Megazyme method allows to assess the total content of branched and non-branched glucan particles. The method consists of the hydrolysis of polysaccharides to the basic d-glucose molecules [14,62]. However, enzymatic methods do not allow for a separate evaluation of the content of triple-helix β-glucans. Therefore, for these compounds, the method of triple extraction with aqueous solutions of alkalia and acids according to Nitschke et al. [29] was used.

### 3.4. Evaluation of β-Glucans in Wild and Commercial Cultivated Mushrooms

In our research, a classification into commercial cultivated and wild mushrooms was adopted in the literature; the most studied species for β-glucan content are commercial cultivated mushrooms the *Pleurotus*, *Lentinula* and *Agaricus* genus, mainly due to the good accessibility of the test material. Among the *Pleurotus* genus, *Pleurotus ostreatus, Pleurotus eringa, Pleurotus djamor Pleurotus ferule, Pleurotus nebrodensis*, and *Pleurotus sapidus,* popular in China, Korea and Japan but less known in Poland, were studied as well. Among the *Lentinula* genus, the most studied is *Lentinula edodes*, and among the *Agaricus* genus, different varieties of *Agaricus bisporus* [31,43,66].

The literature data on β-glucans in wild mushrooms is limited. Our research (Table 1, Table 2) and that of other authors [29,63,67] indicates a β-glucan content in wild mushrooms comparable to cultivated species.

Basic research, which determines the amount of biologically active components in different mushrooms species allows for the selection of species that have a significant content of β-glucans, thus indicating mushrooms whose cultivation for commercial purposes (food and pharmaceutical industries) can be important. Although there are many reports on the biological activity and structure of β-glucans of fungal origin [68,69,70], there are few publications on the quantitative evaluation of these compounds in wild mushrooms [9,29].

Earlier studies have shown that β-glucans are the leading biologically active compounds of medicinal mushrooms [64,71]. It was found that the β-glucan content in the fruiting bodies of mushrooms grown in a controlled environment [33,43], as well as on specimens from the natural environment [67], may depend on the species, substrate components, environmental conditions and even mycelium strains obtained from various in vitro cultures [31,56]. Therefore, we studied wild mushrooms to compare them with cultivated species. A comparable content of β-glucans assayed by the Megazyme method in wild and cultivated mushrooms was found, and a higher content (but without confirmed statistical significance) of β-glucans determined by Congo red method in wild grown mushrooms was found (Table 3).

In this study, we found that the wild mushrooms with the highest content of β-glucans determined with the Congo red method were *Lactarius deliciosus* (17.11 g/100 g DM) and *Suillus grevillei* (16.96 g/100 g DM). The content of these compounds was slightly higher compared to mushrooms of proven medical importance (*Pleurotus ostreatus* −12.39 g/100 g DM) (Table 2) and significantly higher compared to other medicinal species: *Agaricus bisporus* (3.12 g/100 g DM) and *Lentinula edodes* (8.42 g/100 g DM) (Table 2).

At the same time, according to our knowledge, there is no literature data on the content of triple-helix β-glucans in the fruiting bodies of the *Suillus* and *Lactarius* genus. However, there are several publications available confirming the therapeutic properties of polysaccharides with the structure of β-glucans isolated from *Lactarius deliciosus* [72,73]. The content of β-glucans determined by the Congo red method in wild and commercial cultivated mushrooms in our study is similar to the results of other authors [29,73,74].

The high β-glucan content of the genus *Lactarius* was also confirmed by Ruthes et al. [72] and Hou et al. [73]. Mushrooms of the genus *Russula* are rarely assessed for their β-glucan content; however, a few scientific reports confirm the high β-glucan content in their cell walls compared to other mushrooms [30,67,75].

Some of the examined mushrooms species were characterized by a significantly lower content of β-glucans determined with the Congo red method. These were mainly *Cantharellus cibarius* (1.946 g/100 g DM), *Agaricus bisporus* (3.117 g/100 g DM), *Xerocomellus chrysenteron* (5.095 g/100 g DM), *Boletus subtomentosus* (5.138 g/100 g DM), *Macrolepiota procera* (5.461 g/100 g DM) and *Boletus edulis* (5.801 g/100 g DM). Similar results for *Boletus edulis* were obtained by Boonyanuphap et al. [67], who studied the β-glucan content of 32 species of fungi from the forest areas of Thailand [67]. Similar relationships between β-glucans determined with the Congo red in the mycelium and fruiting bodies of mushrooms were observed by Nitschke et al. [29]. Nevertheless, the low content of β-glucans in Boletus edulis does not diminish its health promoting values, which result from the favorable proportions of nutrients, vitamins, minerals, and biologically active compounds other than β-glucans, such as polyphenols, ergosterol, indole compounds or microelements [36,76,77,78,79,80,81,82].

The β-glucan content determined with the Congo red method of several wild mushrooms (*Tricholoma portentosum, Suillus bovinus, Russula heterophylla, Russula vinosa, Suillus grevillei* and *Lactarius deliciosus*) exceeded the content of these compounds in the mushroom species considered as medicinal (*Agaricus bisporus, Lentinula edodes* and *Pleurotus ostreatus*) (Table 2, Figure 2). However, the differences in β-glucan content in these groups did not reach statistically significant levels (Table 3).

The content of β-glucans in the fruiting bodies of commercial species with proven medicinal properties largely depends on the growing conditions, the type of substrate and the mycelium strain used. This allows to control to some extent the content of these compounds in the final product [33,83]. Few publications report on experiments to enrich the fruiting bodies of cultivated mushrooms with β-glucans. In the research of Park et al. [33], whose aim was to obtain cultivated mushrooms with a higher content of β-glucans, a solution of enzymes (chitinase, β-glucuronidase, and other lytic enzymes) was applied to the mycelium and the fruiting bodies of *Sparassis* mushrooms, which initiated elicitation in the cell walls. This reaction in turn contributed to an increase in β-glucan content in strains of cultivated fungi of the *Sparassis crispa* species (now *Sparassis latifolia*). The authors proposed the use of elicitation as a method of obtaining edible mushrooms of greater medical significance [33]. The β-glucans in the fruiting bodies of the cauliflower fungus (*Sparassis crispa*) has been the subject of Japanese studies [84]. The results obtained by the Japanese team confirm the therapeutic properties of the *Sparassis crispa* in oral use. It has been shown that the main component responsible for the properties of this species are β-glucans, the content of which in the studied fruiting bodies of mushrooms was determined to be more than three times higher [84] compared to the content of β-glucans in *Pleurotus ostreatus* from own research (Table 2). The main β-glucan isolated from the cauliflower fungus was SCG β-glucan (*Sparassis crispa* glucan) [84].

According to current scientific knowledge, mushroom β-glucans, especially triple-helix β-glucans, represent a promising therapeutic way in anti-tumor therapy due to low side effects [85], in allergy therapy [85] and others, like wound healing therapy [27]. Furthermore, beta-glucans can be used in vaccines [86] and in nanotechnology and microencapsulation [87]. Moreover, the most recent research shows that β-glucan extracts from edible mushrooms have coronavirus disease (COVID-19) alleviating properties by reduction of pro-inflammatory cytokines and oxidative stress [88].

## 4. Conclusions

In this paper, we report on the content of β-glucans determined with two methods, the Megazyme method and the Congo red method, which allows to determine β-glucans with triple-helix chain conformation [29]. β -glucans were studied in 18 species of wild edible Polish fungi, with the aim of comparing them with commercial mushrooms and making comparisons between the species. Furthermore, the percentage difference in β-glucans assayed by the Congo red method and the Megazyme method was determined, as well as quantitative evaluation of β-glucans in acidic and alkaline pH extracts in Congo red method was performed.

According to our knowledge, this is the first study that evaluates the content of β-glucans by two methods, the Megazyme method and the Congo red method in 21 edible mushroom species including 18 wild mushroom species. We found that the highest content of β-glucans determined by the Megazyme method, among wild mushrooms, was in *Tricholoma portentosum* (34.97 g/100 g DM), whereas β-glucans determined by the Congo red method in *Lactarius deliciosus* (17.11 g/100 g DM) and *Suillus grevillei* (16.97 g/100 g DM). This study found that wild mushrooms have a comparable or higher content of β-glucans compared to commercially grown species with proven medicinal properties, namely *Agaricus bisporus*, *Lentinula edodes* or *Pleurotus ostreatus*.

This study indicates wild mushrooms as an interesting material for β-glucan extraction.

However, further research is needed on the quantitative content, molecular structure, and conformation of glucans in wild mushrooms, which will indicate further possibilities for their wider application in food production, pharmacology and medicine.

## Figures and Tables

**Table 1:** Glucan (total glucans, alpha-glucans and beta-glucans) and moisture content in wild grown and commercial cultivated mushrooms, assayed with the Megazyme assay kit.

No.	Mushroom Species	*N*	Moisture %	Total Glucans *		Alpha–Glucans **		Beta-Glucans ***	
				X ± SD	Range	X ± SD	Range	X ± SD	Range
				g/100 g DM					
**wild edible mushrooms**									
1.	*Armillaria mellea* (Vahl) P. Kumm.	3	86.57–91.76	26.2 ± 2.92	23.1–28.9	1.11 ± 0.08	1.05–1.20	25.09 ± 2.85	22.05–27.70
2.	*Imleria badia* (Fr.) Fr.	4	89.23–93.55	27.7 ± 0.38	27.4–28.1	3.24 ± 1.38	1.82–4.58	24.43 ± 1.05	23.52–25.58
3.	*Boletus edulis* Bull.	3	90.72–92.04	31.0 ± 0.87	30.0–31.5	3.90 ± 1.87	2.20–5.90	27.10 ± 1.95	25.60–29.30
4.	*Boletus subtomentosus* L.	3	88.39–92.62	22.4 ± 3.44	19.8–26.3	4.10 ± 3.13	1.35–7.50	18.30 ± 1.75	16.35–19.75
5.	*Cantharellus cibarius* Fr.	4	91.50–93.17	27.4 ± 1.01	26.5–28.3	1.40 ± 0.22	1.11–1.64	25.98 ± 1.20	24.86–27.09
6.	*Lactarius deliciosus* (L.) Pers.	3	90.79–92.11	29.4 ± 0.38	29.1–29.8	1.74 ± 0.68	1.28–2.52	27.62 ± 0.30	27.28–27.77
7.	*Leccinum aurantiacum* (Bull.) Gray);	3	90.50–91.53	32.9 ± 3.45	30.3–36.8	5.80 ± 2.79	3.44–8.88	27.06 ± 5.59	22.62–33.34
8.	*Leccinum scabrum* (Bull.) Gray)	3	83.91–91.84	26.8 ± 2.94	23.6–29.4	3.05 ± 1.46	1.68–4.58	23.71 ± 2.45	21.92–26.50
9.	*Macrolepiota procera* (Scop.) Singer.	3	82.00–87.06	11.4 ± 2.34	10.0–11.4	0.90 ± 0.58	0.23–1.25	10.50 ± 0.30	8.89–12.85
10.	*Rozites caperatus* (Pers.) P. Karst.	4	91.15–93.26	33.6 ± 1.20	32.4–34.8	0.79 ± 0.24	0.51–0.95	32.78 ± 1.41	31.49–34.29
11.	*Russula heterophylla* (Fr.) Fr.	3	92.50–94.83	35.6 ± 1.85	33.5–37.0	8.57 ± 2.64	6.40–11.50	27.03 ± 1.50	25.50–28.50
12.	*Russula vinosa* Lindblad	3	86.27–90.36	31.8 ± 0.30	31.5–32.1	7.56 ± 4.13	2.80–10.20	24.24 ± 4.12	21.83–29.00
13.	*Suillus bovinus* (Pers.) Roussel	3	82.91–85.73	28.4 ± 0.14	28.3–28.5	1.83 ± 0.28	1.52–2.06	26.57 ± 0.52	26.24–26.98
14.	*Suillus grevillei* (Klotzsch) Singer	3	92.38–95.73	27.9 ± 1.06	27.1–29.0	0.70 ± 0.08	0.65–0.79	27.15 ± 1.11	26.22–28.38
15.	*Suillus luteus* (L.) Roussel	3	91.32–94.22	29.7 ± 2.08	28.4–32.1	1.48 ± 0.06	1.42–1.54	28.22 ± 2.14	26.86–30.68
16.	*Tricholoma equestre* (L.) P. Kumm.	3	86.30–92.09	24.2 ± 1.62	23.3–26.1	0.96 ± 0.53	0.55–1.56	23.27 ± 2.01	21.74–25.55
17.	*Tricholoma portentosum* (Fr.) Quel.	3	91.59–93.36	36.9 ± 1.06	35.7–37.7	1.93 ± 0.15	1.80–2.10	34.97 ± 1.21	33.60–35.90
18.	*Xerocomellus chrysenteron* (Bull.) Šutara	3	93.44–94.38	15.5 ± 2.62	11.3–16.5	1.35 ± 0.33	1.00–1.65	12.72 ± 2.94	9.65–15.50
**commercial cultivated mushrooms**									
19.	*Lentinula edodes* (Berk.) Pegler	3	90.94–90.76	26.7 ± 3.22	24.74–30.40	0.42 ± 0.05	0.38–0.47	26.26 ± 3.23	24.74–30.40
20.	*Pleurotus ostreatus* (Jacq.) P. Kumm.	4	90.93–91.91	45.9 ± 1.62	45.0–47.80	5.59 ± 0.62	5.09–6.29	40.34 ± 3.23	39.61–41.51
21.	*Agaricus bisporus* (J.E. Lange) Imbach)	3	91.48–92.54	15.8 ± 3.47	12.60–19.50	4.47 ± 2.04	2.41–6.48	11.36 ± 2.85	8.07–13.02

DM, dry mass; No., number of mushroom species; SD, standard deviation; X, mean; * Total glucans (α-glucan +β-glucan) + d-glucose in oligosaccharides, sucrose and free d-glucose; ** alpha-glucans + d-glucose in sucrose and free d-glucose; *** Beta-glucans—calculated by difference between total glucans with d-glucose in oligosaccharides, sucrose and free d-glucose and alpha-glucans including d-glucose in sucrose and free d-glucose. Calculations were performed according to manufacturer’s instructions.

**Table 2:** The Congo red positive β-glucans in the studied wild and commercial mushrooms in different fractions.

No.	Mushroom Species	*N*	β-Glucans	KOH β-Glucans Fraction a	HCl β-Glucans Fraction b	NaOH β-Glucans Fraction c
			X ± SD g/100 g DM			
**wild edible mushrooms**						
1.	*Armillaria mellea* (Vahl) P. Kumm.	3	12.156 ± 1.751	6.205 ± 1.168	2.133 ± 0.206	3.818 ± 0.418
2.	*Imleria badia (Fr.) Fr.*	4	9.318 ± 1.871	5.214 ± 1.294	1.551 ± 0.185	2.615 ± 0.418
3.	*Boletus edulis* Bull.	3	5.801 ± 1.217	3.583 ± 1.097	1.114 ± 0.158	1.105 ± 0.313
4.	*Boletus subtomentosus* L.	3	5.138 ± 1.041	1.797 ± 0.650	1.099 ± 0.075	2.242 ± 0.489
5.	*Cantharellus cibarius* Fr.	4	1.946 ± 0.182	ND	1.010 ± 0.085	0.936 ± 0.103
6.	*Lactarius deliciosus* (L.) Pers.	3	17.110 ± 1.708	7.423 ± 0.692	3.037 ± 0.390	6.649 ± 0.732
7.	*Leccinum aurantiacum* (Bull.) Gray	3	8.085 ± 2.041	2.842 ± 0.941	1.287 ± 0.303	3.956 ± 0.831
8.	*Leccinum scabrum* (Bull.) Gray	3	5.908 ± 0.585	2.213 ± 0.645	1.110 ± 0.192	2.585 ± 0.038
9.	*Macrolepiota procera* (Scop.) Singer.	3	5.461 ± 0.601	1.523 ± 0.305	1.580 ± 0.017	2.357 ± 0.420
10.	*Rozites caperatus* (Pers.) P. Karst.	4	10.026 ± 3.377	3.970 ± 1.653	1.544 ± 0.530	4.512 ± 1.527
11.	*Russula heterophylla* (Fr.) Fr.	3	14.347 ± 0.812	6.765 ± 0.587	2.929 ± 0.260	4.653 ± 0.620
12.	*Russula vinosa* Lindblad	3	14.665 ± 0.756	5.427 ± 0.459	3.063 ± 0.533	6.176 ± 0.444
13.	*Suillus bovinus* (Pers.) Roussel	3	13.299 ± 0.651	9.509 ± 0.642	0.926 ± 0.188	2.864 ± 0.458
14.	*Suillus grevillei* (Klotzsch) Singer	4	16.965 ± 1.694	7.810 ± 1.150	2.744 ± 0.207	6.411 ± 0.570
15.	*Suillus luteus* (L.) Roussel	3	12.149 ± 1.043	8.479 ± 1.277	1.190 ± 0.169	2.480 ± 0.312
16.	*Tricholoma equestre* (L.) P. Kumm.	3	10.323 ± 1.273	3.269 ± 0.630	1.877±	5.178 ± 0.066
17.	*Tricholoma portentosum* (Fr.) Quel.	3	12.912 ± 1.546	5.391 ± 1.136	2.394 ± 0.147	5.127 ± 0.475
18.	*Xerocomellus chrysenteron* (Bull.) Šutara	3	5.095 ± 1.275	3.034 ± 1.302	0.644 ± 0.005	1.417 ± 0.035
**commercial mushrooms**						
19.	*Pleurotus ostreatus* (Jacq.) P. Kumm.	4	12.393 ± 1.100	4.698 ± 0.734	1.709 ± 0.217	5.986 ± 0.365
20.	*Agaricus bisporus* (J.E. Lange) Imbach)	3	3.117 ± 0.101	0.879 ± 0.070	1.106 ± 0.049	1.132 ± 0.108
21.	*Lentinula edodes* (Berk.) Pegler	3	8.417 ± 0.627	3.894 ± 0.523	1.005 ± 0.044	3.518 ± 0.147

DM, dry mass; ND, not detected; SD, standard deviation; β-glucans- a+ b+ c; X, mean.

**Table 3:** β-Glucans content in wild mushrooms vs. commercial mushrooms.

Glucans	Wild Grown *N* = 58		Commercial Cultivated *N* = 10		*p*
	g/100 g DM				
	The Megazyme Method				
β-glucans	Q1	Q3	Q1	Q3	─
	22.52	27.77	13.02	39.61	─
	Me		Me		─
	26.23		27.25		0.516
	X ± SD		X ± SD		─
	25.01 ± 6.05		27.34 ± 12.76		─
**The Congo red method**					
β-glucans (KOH fraction)	Q1	Q3	Q1	Q3	─
	2.36	6.65	0.96	4.39	─
	Me		Me		─
	4.34		4.06		0.156
	X ± SD		X ± SD		─
	4.66 ± 2.71		3.311 ± 1.78		─
β-glucans (HCl fraction)	Q1	Q3	Q1	Q3	─
	1.06	2.45	1.05	1.68	─
	Me		Me		─
	1.51		1.13		0.236
	X ± SD		X ± SD		─
	1.72 ± 0.80		1.32 ± 0.36		─
β-glucans (NaOH fraction)	Q1	Q3	Q1	Q3	─
	2.12	5.10	1.24	5.86	─
	Me		Me		─
	3.13		3.59		0.736
	X ± SD		X ± SD		─
	3.60 ± 1.85		3.79 ± 2.14		─
β-glucans (KOH fraction + HCl fraction + NaOH fraction)	Q1	Q3	Q1	Q3	─
	6.05	13.68	3.20	11.82	─
	Me		Me		─
	10.13		8.75		0.295
	X ± SD		X ± SD		─
	10.02 ± 4.57		8.41 ± 4.11		─

Me, median; Q, quartile; X, mean; SD, standard deviation.
